# Away from darkness: a review on the effects of solar radiation on heterotrophic bacterioplankton activity

**DOI:** 10.3389/fmicb.2013.00131

**Published:** 2013-05-23

**Authors:** Clara Ruiz-González, Rafel Simó, Ruben Sommaruga, Josep M. Gasol

**Affiliations:** ^1^Department of Marine Biology and Oceanography, Institut de Ciències del Mar, CSICBarcelona, Spain; ^2^Département des Sciences Biologiques, Université du Québéc à MontréalMontréal, QC, Canada; ^3^Laboratory of Aquatic Photobiology and Plankton Ecology, Institute of Ecology, University of InnsbruckInnsbruck, Austria

**Keywords:** solar radiation, aquatic ecosystems, bacterioplankton community composition, bacterial heterotrophic activity, light history

## Abstract

Heterotrophic bacterioplankton are main consumers of dissolved organic matter (OM) in aquatic ecosystems, including the sunlit upper layers of the ocean and freshwater bodies. Their well-known sensitivity to ultraviolet radiation (UVR), together with some recently discovered mechanisms bacteria have evolved to benefit from photosynthetically available radiation (PAR), suggest that natural sunlight plays a relevant, yet difficult to predict role in modulating bacterial biogeochemical functions in aquatic ecosystems. Three decades of experimental work assessing the effects of sunlight on natural bacterial heterotrophic activity reveal responses ranging from high stimulation to total inhibition. In this review, we compile the existing studies on the topic and discuss the potential causes underlying these contrasting results, with special emphasis on the largely overlooked influences of the community composition and the previous light exposure conditions, as well as the different temporal and spatial scales at which exposure to solar radiation fluctuates. These intricate sunlight-bacteria interactions have implications for our understanding of carbon fluxes in aquatic systems, yet further research is necessary before we can accurately evaluate or predict the consequences of increasing surface UVR levels associated with global change.

## Introduction

Solar radiation supplies the energy necessary for the functioning of planktonic communities, either directly through the action of biologically usable photons, or indirectly by driving fluid motion and feeding and shaping the reducing power of organic matter (OM). However, part of sunlight energy occurs at wavelengths that can harm the biota of the surface ocean and freshwaters; this is mainly the case of the ultraviolet region of the spectrum (UVR, 280–400 nm). Bacterioplankton, in particular, are considered amongst the most sensitive organisms to UVR-induced damage owing to their general lack of pigmentation and low internal self-shading due to small cell volume (García-Pichel, 1994; Jeffrey et al., 1996a). In aquatic ecosystems, bacteria play a central role in the cycling of nutrients and the energy flow to higher trophic levels, transforming and consuming most of the OM (Azam et al., 1983; Cole et al., 1988; Ducklow, 2000). Since, at least in the ocean, about one half of the total prokaryotic heterotrophic production is concentrated in the thin sunlit surface layer (Arístegui et al., 2009), it is likely that any sunlight-driven effect on them will influence the amount of OM respired or channeled throughout the microbial food web.

Research on the effects of sunlight on the heterotrophic activity of natural bacterial communities over the past 30 years has revealed that, contrary to what was initially believed, sunlight is not always detrimental. A wide range of positive and negative effects are found throughout the literature, yet the reasons underlying the ultimate (observed) effect and its variability are not fully understood. Since the published studies cover a wide array of light characteristics, seasons, latitudes, depths, physico-chemical conditions, and experimental designs, inferences of general trends in the bacterial response to sunlight, or predictions of the role of this environmental factor in modulating bacterial biogeochemical functions in aquatic systems, are not straightforward.

The few published reviews on the effects of sunlight on bacterial activity (which mainly referred to UVR only) date from the early 2000's (Jeffrey et al., 2000; Moran and Zepp, 2000) and have become outdated after the large number of studies that have recently been published (see Table A1 in Appendix). Indeed, new perspectives have been opened by the discovery of bacterial light harvesting mechanisms other than photosynthesis (Béjà et al., 2000; Kolber et al., 2000), and the development and use of single-cell approaches has unveiled a significant diversity in taxon-specific bacterial responses to sunlight, with implications for light-driven changes at the community level (Alonso-Sáez et al., 2006; Straza and Kirchman, 2011; Ruiz-González et al., 2012e). In addition, new approaches including detailed measurements of water column irradiance and devices that simulate mixing have provided novel insights into the role that the previous light exposure conditions play in microbial dynamics (Bertoni et al., 2011; Ruiz-González et al., 2012e). Finally, the majority of studies still draw conclusions from occasional or even single experiments, even though there is experimental evidence that bacterial responses are modulated by the time scales at which exposure to sunlight varies.

This recent body of knowledge, though, has neither been integrated into a wider framework nor explored for its ecological and biogeochemical implications. Although there are some very recent reviews on the effects of UVR (but not PAR) on aquatic biota (Häder et al., 2011; Llabrés et al., 2013), they only briefly touch upon its specific impact on bacterial heterotrophic activity. Similarly, increasing scientific interest on light-harvesting prokaryotes has motivated reviews focused on photoheterotrophic bacteria, yet ignoring the functioning of these organisms under full sunlight conditions (Moran and Miller, 2007; Fuhrman et al., 2008; Zubkov, 2009).

In this review, we compile the existing literature on the effects of natural levels of light (natural or simulated PAR and UVR) on bacterioplankton heterotrophic activities, examining all the potential causes for the observed diversity of responses, with special emphasis on the largely overlooked roles of bacterial community composition and the previous light exposure conditions. Moreover, we integrate the studies that have attempted to address how the responses of bacteria may vary at different sunlight-relevant temporal and spatial scales. Finally, we will discuss the potential implications of the observed patterns for the measurement of carbon cycling fluxes.

## Sunlight beneath the water surface: wavelength dependent attenuation in the water column

Even though the vast majority of the world oceans' volume is shrouded in darkness, the processes occurring within the thin illuminated surface layer (the photic layer, the upper 50–170 m) are of enormous significance to the global biosphere. For example, the visible region of the solar spectrum (so-called photosynthetically available radiation or PAR) reaching this sunlit layer fuels about half of the primary productivity of the planet, and is thus responsible for roughly half of the atmospheric oxygen necessary for most life on Earth (Walker, 1980; Longhurst et al., 1995).

The spectrum of the solar radiation striking the Earth's surface spans from ca. 290 nm to about 2500 nm, and can be divided into different regions of increasing wavelength: the ultraviolet radiation (UVR, 280–400 nm), the visible light or PAR (400–700 nm), and the infrared radiation (>700 nm). The UV region is classified into two wavelength ranges, UVA (320–400 nm), and UVB (280–320 nm), the latter being considered the most biologically harmful fraction of the solar spectrum per photon unit. The UVC range (100–280 nm) is entirely absorbed in the atmosphere and thus does not reach the Earth's surface. The loss of sunlight in the water column begins with reflection at the surface, whose magnitude varies depending on solar elevation and surface roughness. Once beneath the surface, sunlight attenuation is a function of wavelength: in general, blue light (450–495 nm) penetrates the deepest (but see Eloranta, 1978), attenuation sharply increasing toward shorter wavelengths [through violet (400–450 nm) and UVA to UVB], and toward longer wavelengths [through green (495–570 nm) to infrared]. As a result, deeper waters are enriched in blue light, and the relative ratios of UVB to UVA or short wavelength PAR decrease with depth (Díaz et al., 2000; Hargreaves, 2003).

Besides the optical properties of the water molecules (see Boss et al., 2007), several other factors influence the depth of sunlight penetration: colored dissolved organic material (CDOM) absorbs short wavelengths, phytoplankton pigments absorb visible light, and suspended particles scatter and absorb throughout the spectrum (Bracchini et al., 2006; Sommaruga and Augustin, 2006). As a consequence, sunlight penetration is usually low in coastal, estuarine, and lowland freshwater ecosystems characterized by high concentrations of CDOM and particles, where UV irradiance is extinguished within the top 10 m of the water column (Tedetti and Sempéré, 2006; Häder et al., 2011; Smyth, 2011). In the open ocean and oligotrophic lakes, conversely, UVR penetrates to a considerable depth, reaching up to 68 m in the clearest waters of the ultraoligotrophic South Pacific gyre (Morel et al., 2007) and lakes of similar transparency (Vincent et al., 1998; Hargreaves et al., 2007).

The preconception that UVR penetration into water was insignificant made early researchers assume that the impacts of sunlight on aquatic biota should be negligible (Jerlov, 1950). However, by the mid 1980's, concerns on the destruction of the stratospheric ozone layer and threats of increases in UVB radiation, together with the discovery that UVR penetrates much deeper into water than previously thought (Worrest and Häder, 1989; Karentz and Lutze, 1990), prompted urgent research to examine its effects on living beings. Since then, multiple studies have demonstrated that UVR can likely affect all the inhabitants of surface waters (see references in Häder, 2011; Llabrés et al., 2013), with potential implications for the cycling of OM in aquatic ecosystems.

## Effects of sunlight intensity and spectrum on bacterioplankton

Amongst all the potential targets of sunlight penetrating the surface waters, heterotrophic bacteria have received particular attention due to their recognized significance in the cycling of carbon and energy (Azam et al., 1983; Cole et al., 1988; Ducklow, 2000). Pioneering studies on the effects of solar radiation on bacteria date back to the nineteenth century, when Downes and Blunt (1877) reported that sunlight exposure precluded the growth of bacteria in different media, and argued that this bactericidal action was dependent on the intensity, duration, and wavelength within the sunlight spectrum. Other early bacteriologists also verified the negative effects of natural sunlight on bacteria (Ward, 1894) and, thenceforth, a number of experiments with pure cultures (mainly of coliforms and pathogens) have confirmed and extended these negative effects of light on bacteria under natural or artificial light sources (see references in Hockberger, 2002). Other research, however, minimized the extent of sunlight effects on the assumption of its strong attenuation in water (Zobell and McEwen, 1935; Pearson, 1956), and while most studies focused on the effects of UVR, the effects of visible radiation attracted much less attention.

**Figure 1:**
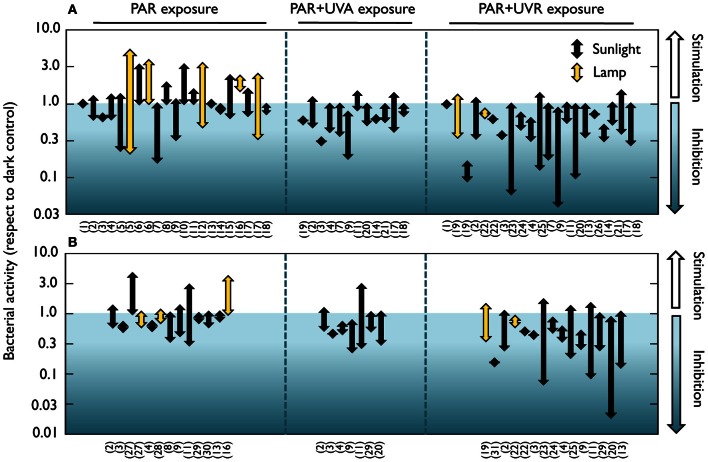
**Positive and negative effects of natural or simulated solar radiation on marine bacterial activity in natural samples.** Range of reported light-driven effects on bacterial heterotrophic activity from different marine systems measured as **(A)** ^3^H-leucine or **(B)** ^3^H-thymidine incorporation rates and expressed as the ratio to dark incubation rates. Whether samples were exposed to natural or simulated radiation (PAR or UVB lamps) is also indicated. Note the logarithmic scales of the ratio on the Y axes. Data were extracted from 31 marine studies in which a dark control was available for comparison with light treatments. Experiments where something else than light was manipulated (e.g., nutrients or temperature) were not considered. (1) Carlucci et al., 1985; (2) Aas et al., 1996; (3) Sommaruga et al., 1997; (4) Visser et al., 1999; (5) Morán et al., 2001; (6) Church et al., 2004; (7) Alonso-Sáez et al., 2006; (8) Church et al., 2006; (9) Hernández et al., 2006; (10) Michelou et al., 2007; (11) Pakulski et al., 2007; (12) Calvo-Díaz, 2008; (13) Joux et al., 2009; (14) Bertoni et al., 2011; (15) del Valle et al., 2012; (16) Ruiz-González et al., 2012c; (17) Ruiz-González et al., 2012e; (18) Ruiz-González et al., 2012f; (19) Herndl et al., 1993; (20) Pakulski et al., 2008; (21) Ruiz-González et al., 2012a; (22) Kaiser and Herndl, 1997; (23) Pakulski et al., 1998; (24) Chróst and Faust, 1999; (25) Visser et al., 2002; (26) Bullock and Jeffrey, 2010; (27) Shiah, 1999; (28) Renaud et al., 2005; (29) Conan et al., 2008; (30) Rochelle-Newall et al., 2008; (31) Müller-Niklas et al., 1995.

**Figure 2:**
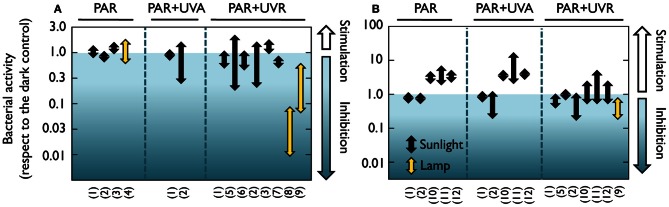
**Positive and negative effects of natural or simulated solar radiation on freshwater and estuarine bacterial activity in natural samples.** Range of reported light-driven effects on bacterial heterotrophic activity from different freshwater and estuarine systems measured as **(A)** ^3^H-leucine or **(B)** ^3^H-thymidine incorporation rates and expressed as the ratio to dark incubation rates. Whether samples were exposed to natural or simulated radiation (visible light or UVB lamps) is also indicated. Data extracted from 12 freshwater or estuarine studies in which a dark control was available for comparison with light treatments. Experiments where something else than light was manipulated (e.g., nutrients or temperature) were not considered. Note the logarithmic scales of the ratio on the Y axes. (1) Aas et al., 1996; (2) Sommaruga et al., 1997; (3) Ziegler and Benner, 2000; (4) Straza and Kirchman, 2011; (5) Amon and Benner, 1996; (6) Lindell and Edling, 1996; (7) Bullock and Jeffrey, 2010; (8) Santos et al., 2011a; (9) Santos et al., 2012b; (10) Carrillo et al., 2002; (11) Medina-Sánchez et al., 2002; (12) Medina-Sánchez et al., 2006.

These earlier studies employed cultured strains and culture-dependent techniques such as plate counts, but it is now well-known that the microorganisms retrieved by these traditional techniques are not representative of the ecologically relevant aquatic bacteria (e.g., Giovanonni and Rappé, 2000). It was not until the early 80's that the effect of solar radiation on natural bacterioplankton communities was assessed by means of culture-independent methods. Using a combination of autoradiography and epifluorescence microscopy, a reduction in the number of bacteria taking up radioactive amino acids was detected in estuarine waters, and was attributed not only to UVB, but also to UVA and PAR (Bailey et al., 1983). Similarly, UVA delayed the growth of surface marine bacteria (Sieracki and Sieburth, 1986). Conversely, neither visible light nor UVR had any detrimental effect on bacterial incorporation of ^3^H-leucine and ^3^H-glutamic acid in seawater surface films (Carlucci et al., 1985). Later on, Herndl et al. (1993) demonstrated a negative effect of full sunlight exposure on several extracellular enzymatic activities at the sea surface, as well as a clear negative relationship between UVB doses and the incorporation of the two radioactive tracers most commonly used for measuring bacterial production, namely ^3^H-leucine and ^3^H-thymidine (Fuhrman and Azam, 1980; Kirchman et al., 1985). Since then, a rising number of studies have examined the effects of natural or simulated light on bacterial heterotrophic activity, uncovering a remarkable variability among the reported results. A careful look at the light-driven bacterial activity responses throughout the existing literature (Figures 1, 2) unveils effects ranging from total inhibition by UVB (Santos et al., 2011a) up to 150-fold stimulation under PAR + UVA exposure (Medina-Sánchez et al., 2002). Despite this great variability, though, a tendency for a large stimulation due to PAR and maximum inhibition caused by full sunlight exposure is evident at least in marine waters (which have been much more intensely studied than freshwater ecosystems, Figure 1). Ignoring the differences in sites, conditions, and experimental protocols among studies, marine experiments report an average maximum reduction of leucine and thymidine incorporation by UVR of 62 and 71%, respectively, as compared to dark incubations. In contrast, PAR exposure causes a mean maximum stimulation of 100 and 65% in leucine and thymidine incorporation, respectively (Figures 1A,B). It is also interesting to note that significant (or even dominant) inhibition due to PAR and UVA has sometimes been reported (Aas et al., 1996; Sommaruga et al., 1997; Morán et al., 2001; Pakulski et al., 2007). This diversity in the observed responses questions the early assumption that the effects of sunlight on bacteria, if any, should always be detrimental, and suggests that the interplay among sunlight, OM, and aquatic microorganisms is far from simple.

Although less studied, the bacterial extracellular enzymes responsible for cleaving and processing high molecular weight DOM (Chróst, 1992) are also known to be photochemically degraded, with implications for nutrient regeneration in aquatic systems. The available studies indicate that ectoenzyme activities are negatively impacted by PAR and UVR irradiation (Herndl et al., 1993; Müller-Niklas et al., 1995; Garde and Gustavson, 1999; Espeland and Wetzel, 2001; Santos et al., 2011a), albeit some PAR-driven photostimulation (Ruiz-González et al., 2012f), and even some UVR-driven reactivation of activity (Boavida and Wetzel, 1998) have also been reported.

## Causes of variability in bacterial responses to sunlight

**Figure 3:**
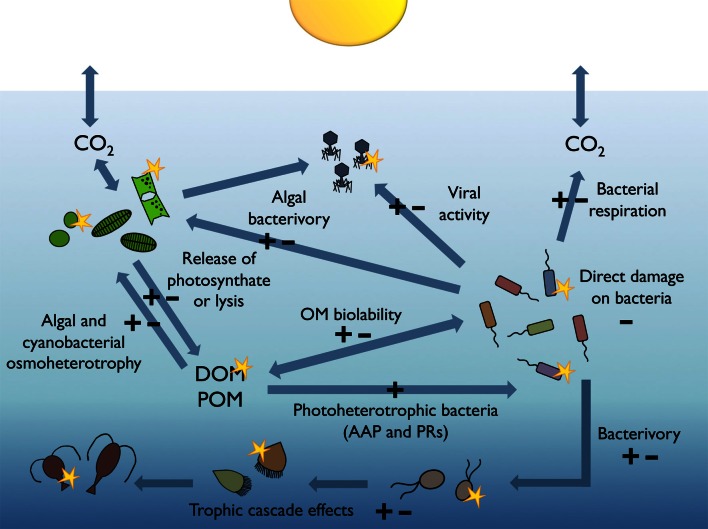
**Sunlight-modulated interactions among microbes and molecules.** Simplified scheme of the pelagic marine food web illustrating the processes susceptible to be modulated by solar radiation either positively (+) or negatively (−), which may ultimately lead to increases or decreases in the heterotrophic activity of bacterioplankton.

As depicted by Figures 1, 2, either inhibition or stimulation of bacterial heterotrophic activities upon exposure to sunlight has been observed. This is because direct damage is only one of the mechanisms explaining bacterial responses to sunlight; the taxonomic composition of the bacterial assemblage, their acclimation to sunlight, the availability, production, and characteristics of OM, and the sunlight susceptibility of competitors, bacterivores, and phages, may all interact to drive the observed responses of bacteria to irradiation (Figure 3). In addition, other environmental factors such as nutrient availability, temperature, and water mixing significantly contribute to modulate the interplay between bacteria and sunlight. Here, we will briefly examine these potential mechanisms one by one.

### Direct sunlight damage of the cellular machinery and mechanisms of repair

Damage by UVB mainly results from direct photon absorption by molecules, mostly DNA and proteins (Setlow, 1960; Jagger, 1985; Tyrrell, 1986). The effects of UVA, instead, are mostly indirect, and occur through the formation of reactive oxygen species-ROS (Harrison, 1967), which cause oxidative damage to several cellular targets including DNA, RNA, proteins, and lipids. Even though UVB is more biologically harmful than UVA on a per photon basis, the higher photon fluxes and deeper penetration of UVA into the water column make it a stronger source of biological damage (Karentz et al., 1994; Sommaruga et al., 1997). The harmful effects associated with PAR are also attributed to photodynamic processes involving ROS (Harrison, 1967).

In spite of their lack of efficient physical protection from solar radiation (e.g., García-Pichel, 1994), bacteria are capable of coping with high irradiances by readily repairing the damage caused during exposure (Jeffrey et al., 1996a,b; Kaiser and Herndl, 1997). DNA damage can be reversed by three different mechanisms: photoenzymatic repair (PER), nucleotide excision repair, and postreplication repair. While the latter two (so-called “dark repair systems”), require ATP and can operate both in the dark and the light (Friedberg, 1985; Sancar and Sancar, 1988), the expression of the photolyase enzyme responsible for PER requires long-wavelength UVA and short-wavelength PAR (Kim and Sancar, 1993). It has recently been proposed that UVB can also induce photolyase expression (Matallana-Surget et al., 2009), thus further stressing the dual function of short-wavelength light, which poses the cell at the center of a balance between damage induction and repair rates that will determine the final rate of damage accumulation.

### Indirect effects of sunlight on bacterial heterotrophic activity

Besides the direct absorption of photons by cells, sunlight modulates many other processes that might ultimately lead to changes in bacterioplankton heterotrophic activity. For example, much non-living OM undergoes photochemical alterations upon absorption of different regions of the solar spectrum. Depending on the quality of DOM, sunlight can either photolyse some recalcitrant DOM into more readily utilizable forms, thus enhancing the activity of heterotrophic bacteria, or instead render initially bio-labile DOM into more recalcitrant compounds (Herndl et al., 1997; Benner and Biddanda, 1998; Chróst and Faust, 1999; Obernosterer et al., 1999, 2001; Biddanda and Cotner, 2003; Kaiser and Sulzberger, 2004; Abboudi et al., 2008; Bastidas-Navarro et al., 2009). UVR has also the potential to transform DOM into inorganic photoproducts, some of which are substrates for bacterial growth (Miller and Zepp, 1995; Amon and Benner, 1996; Perez and Sommaruga, 2007). In addition, UV and visible light can cause the release of DOM from particulate OM (POM) via a largely overlooked process, named photodissolution, which may increase the lability of substrates available for bacteria (Mayer et al., 2006). UVR action on DOM can produce ROS with the aforementioned potential for cellular damage (Mopper and Kieber, 2000; Kaiser and Sulzberger, 2004). Finally, although some authors (Sommaruga et al., 1997) had suggested that photodegradation of ^3^H-leucine or ^3^H-thymidine could occur, the susceptibility of these two extensively used substrates to natural UVR doses was recently experimentally rebutted (Vaughan et al., 2010).

Primary producers provide much of the organic substrate for heterotrophic bacterioplankton, and this supply is also influenced by exposure to sunlight. Several authors attributed the observed light-driven enhancements of ^3^H-leucine or ^3^H-thymidine incorporation rates to increases in the total supply of dissolved organic substrates from phytoplankton, although they did not specifically quantify the photosynthate release (Aas et al., 1996; Shiah, 1999; Medina-Sánchez et al., 2002). Exposure to UVR is known to augment the excretion of organic carbon from stressed phytoplankton (e.g., Carrillo et al., 2002), and the reported damage to cell membranes (Llabrés and Agustí, 2006; Agustí and Llabrés, 2007; Llabrés et al., 2010) may likely increase the release of DOM from the dead or dying cells' cytoplasm. However, exposure to UVR has also been shown to reduce photosynthesis (Helbling et al., 2001; Villafañe et al., 2004; Yuan et al., 2007), or to produce a photosynthate of reduced availability for bacteria (Pausz and Herndl, 1999). Furthermore, phytoplankton might indirectly diminish the detrimental effects of UVR on bacteria through shading, although contrasting results have been obtained in experiments that have tested this possibility (Sommaruga et al., 1997; Alonso-Sáez et al., 2006), suggesting dependence on the concentration and absorption characteristics of the phototrophs.

Besides supplying heterotrophs with autotrophically synthesized OM, prokaryotic or eukaryotic phytoplankton are also able to take up and assimilate dissolved organic compounds under particular conditions (e.g., Paerl, 1991; Bronk et al., 2007). This osmoheterotrophic capacity of phytoplankton appears to be modulated by visible and UV light (Rivkin and Putt, 1987; Gómez-Baena et al., 2008; Kamjunke and Tittel, 2008; Ruiz-González et al., 2012b,d); therefore, a proportion of labile DOM in surface waters may be used up by phototrophs and thus diverted from heterotrophic bacteria depending on the sunlight conditions (Ruiz-González et al., 2012b,d). More data are needed to further assess the ecological importance of this bacterioplankton-phytoplankton competition for labile DOM and how this largely overlooked process might influence bacterial production measurements in dark and light conditions.

Bacterivory and viral infection, both potential significant sources of bacterial mortality (e.g., Guixa-Boixareu et al., 1996; Suttle, 2007), can also be impacted by sunlight. Negative effects of UVB and UVA on the bacterivory of heterotrophic flagellates have been reported for single freshwater and marine species (Sommaruga et al., 1996; Ochs, 1997; Ochs and Eddy, 1998). However, the studies examining the consequences of these effects on natural bacterial communities are scarce (Wickham and Carstens, 1998; Chatila et al., 1999, 2001; Sommaruga et al., 1999), and only on a few occasions a clear positive effect on bacterial growth has been observed (Sommaruga et al., 1996). Similarly, studies with mixotrophic algae reported either an increase (Caron et al., 1993; Isaksson et al., 1999; Tittel et al., 2003) or a decrease (Jones and Rees, 1994; Keller et al., 1994) in bacterivory rates with increasing PAR intensity, as well as a decrease under enhanced UVR (Bastidas-Navarro et al., 2011).

Despite a rising number of studies have shown that sunlight is a major cause of viral destruction (Suttle and Cheng, 1992; Noble and Fuhrman, 1997; Jacquet and Bratbak, 2003; Wilhelm et al., 2003; Yuan et al., 2011), to our knowledge no direct evidence is available for an associated enhancement of bacterial abundance or activity in natural waters. In contrast, exposure to surface UVR levels was shown to cause an increase in damaged prokaryotic cells and an accumulation of viruses, probably due to UVR induction of the lytic cycle in lysogenic bacteria (Maranger et al., 2002).

Finally, sunlight can also alter other interactions among aquatic organisms, including parasitic, competitive, and mutualistic interactions (Sommaruga, 2003), which might induce cascading effects throughout the trophic food web with negative or positive consequences for bacteria. As an example, Mostajir et al. (1999) found that UVB reduced the abundance of large ciliates and diatoms, and led to the growth of small heterotrophic flagellates, picophytoplankton, and bacteria, indicating that UVR radiation has the potential to change the structure and dynamics of the pelagic communities and their associated energy and carbon fluxes. On top of the above enumerated mechanisms, the response of bacteria to solar radiation is further modulated by environmental factors with important roles in cell physiology, such as temperature (Bullock and Jeffrey, 2010; but see Vidussi et al., 2011 and Fouilland et al., 2013), or nutrient availability (Morán et al., 2001; Pausz and Herndl, 2002; Medina-Sánchez et al., 2006; Ogbebo and Ochs, 2008; Joux et al., 2009), which further complicate the picture.

All in all, a simultaneous control of all of these potential sources of variation cannot be achieved without an unaffordable degree of experimental complexity. Nonetheless, one must be conscious that the outcomes of light exposure experiments represent a balance among many synergistic and antagonistic effects that may be taking place simultaneously inside the experimental containers. The deeper the knowledge we can attain concerning these interacting processes, the more accurate our interpretation of the obtained results will be.

Other aspects with a recently uncovered large potential to influence the magnitude and sign of the responses of bacterioplankton to different light conditions include the structure of bacterioplankton communities, their previous sunlight exposure, and the different temporal and spatial scales at which the sunlight varies in aquatic systems. Hereafter, we will examine in more detail the role of these tightly interconnected but largely overlooked factors.

## Role of community composition: taxonomically resolved responses to sunlight

Most of the aforementioned studies considered the bacterial assemblage as a “black box,” meaning that differentiation among taxa was not made. However, experiments with isolated strains and the development of single-cell approaches have started to shed light on the fact that within a given bacterial community there may be UVR sensitive and tolerant phylotypes, bacteria with different repair capabilities, and taxa reacting distinctly to the other light-driven processes described above. In addition, the recent discovery of light harvesting mechanisms through which some heterotrophic bacteria may benefit from sunlight energy (see Zubkov, 2009), suggests that the structure (both taxonomic and functional) of a bacterial assemblage strongly determines its bulk responses to sunlight.

We still know very little of how the effects of PAR and UVR on cellular components or activity are distributed within natural bacterioplankton assemblages. Studies with marine and freshwater isolates have evidenced interspecific variability not only in the accumulation of DNA damage (Joux et al., 1999), but also in their viability after exposure, specific activities, repair mechanisms, growth efficiencies, and resistance to oxidative stress (Helbling et al., 1995; Arrieta et al., 2000; Agogué et al., 2005; Dieser et al., 2010; Hörtnagl et al., 2011; Santos et al., 2011b, 2012a; Matallana-Surget et al., 2012). Since most bacteria are not easily cultivable, though, such isolates may be only minor components of natural bacterial assemblages and the observed responses may not be representative of those in natural communities (e.g., Amann et al., 1995).

By means of culture-independent methods, some studies examined the potential role of sunlight in shaping the composition of bacterioplankton communities. Using PCR-denaturing gradient gel electrophoresis (DGGE) analysis based on 16S rDNA, no differences were detected among the composition of the communities from different highly exposed ultraoligotrophic Andean lakes, but a significant correlation between UVR and the proportion of filamentous bacteria was observed (Corno et al., 2009). Exposure of marine bacterial communities to PAR + UVA during either 1–2 days (Winter et al., 2001) or 13–14 days (Piquet et al., 2010) caused only subtle changes in community composition. In contrast, a considerable reduction was observed in the diversity of freshwater and estuarine bacterial communities exposed for only 9 h to simulated UVB (Santos et al., 2011a). Exposure for 4 h to this same artificial radiation also changed significantly the composition of the communities, as assessed by fluorescence *in situ* hybridization (FISH), with respect to the dark controls (Santos et al., 2012b). In experiments testing the effect of light removal during 5–10 days, most bacterial groups exhibited minor responses (Schwalbach et al., 2005). Contrastingly, heterotrophic bacterial community structure described by terminal restriction fragment length polimorphism (T-RFLP) appeared strongly related to PAR conditions in North Pacific waters (Van Mooy et al., 2004) and it varied along a gradient of sunlight exposure created by incubating the same initial lake water samples at different depths during 4–6 days (Langenheder et al., 2006). More recently, an analysis of 16S rDNA gene libraries from microcosms in Bahia Engaño (Patagonia) showed that 8 days of exposure led to clearly differentiated assemblages between samples exposed to full sunlight and those exposed to PAR + UVA or PAR alone (Manrique et al., 2012). In any case, the different experimental designs used in these studies (sample volumes ranging from 1 to 650 L, incubation times from 1 to 15 days, etc.), precludes comparisons and calls for caution when extrapolations are to be made.

However, if we aim at identifying specific activity responses to light within natural assemblages and not just assemblage compositional changes, we need tools that allow for directly coupling the identity and activity of specific microbes in natural communities. In this regard, techniques such as microautoradiography combined with catalyzed reporter deposition-FISH (MAR-CARD-FISH, Alonso and Pernthaler, 2005), flow cytometry cell sorting (Vesey et al., 1994), PCR-DGGE combined with immunocapturing techniques (Kataoka et al., 2009), and NanoSIMS (Secondary Ion Mass Spectrometry, Lechene et al., 2006) combined to identity probes, may be useful tools to understand how the different bacterial groups within natural mixed assemblages react to the same exposure conditions. The few studies that have attempted to do so have unveiled that the changes observed at the community level may be largely influenced by the identity of the occurring organisms and their specific responses to sunlight.

Alonso-Sáez et al. (2006) used MAR-CARD-FISH to uncover differential effects of sunlight on the specific uptake of ^3^H-leucine among the dominant heterotrophic bacterial groups in Mediterranean Sea waters. Interestingly, they observed that the phylogenetic level targeted by the CARD-FISH probe was decisive for the outcome of the experiment, since while *Alphaproteobacteria* were found to be mainly inhibited by UVA exposure, two subgroups within this bacterial class displayed opposite responses, i.e., PAR exposure caused inhibition of the activity of the dominant SAR11 clade, but stimulation of *Roseobacter*.

**Figure 4:**
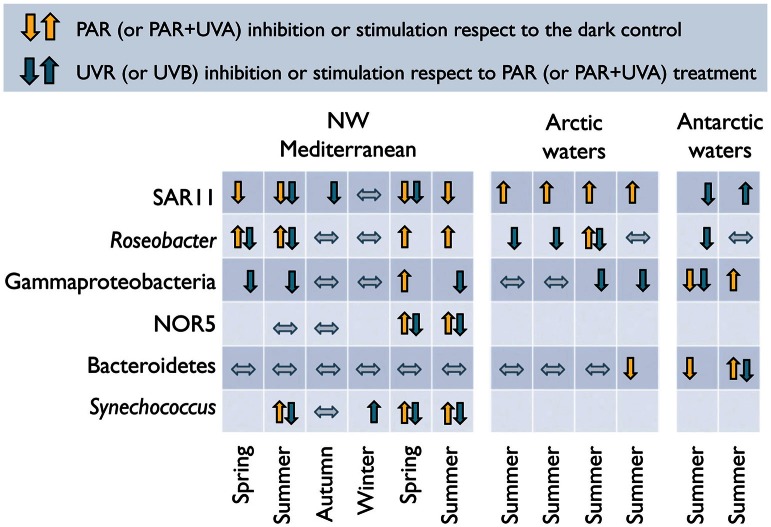
**Diverse responses to sunlight spectrum conditions among different bacterial groups.** Light-driven effects on the percentage of cells active in ^3^H-leucine uptake among different bacterial groups as determined by MAR-CARD-FISH in natural samples. Up- and down arrows indicate significant increase or decrease in the proportion of active cells, respectively, caused by PAR (or PAR + UVA in the case of polar samples, yellow arrows) or full sunlight exposure (or UVB in the case of polar samples, blue arrows). Mediterranean data from Alonso-Sáez et al. (2006) and Ruiz-González et al. (2012f); Arctic and Antarctic values from Ruiz-González et al. (2012a).

Recent analogous experiments in Mediterranean and polar waters have confirmed that these major bacterial taxa display different responses to natural sunlight in terms of ^3^H-leucine or ^35^S-dimethylsulfoniopropionate (^35^S-DMSP) uptake (Ruiz-González et al., 2012a,f), and that these clade-specific responses vary not only with the spectral conditions, but also seasonally and among ecosystems (Figure 4). Interestingly, although broad taxonomic clades (e.g., *Alphaproteobacteria*, *Gammaproteobacteria*, *Bacteroidetes*) include a variety of phylotypes adapted to different conditions (Giovanonni and Rappé, 2000), some consistent patterns can be detected within particular ecosystems. For example, while the Mediterranean SAR11 appear to be particularly sensitive to PAR and UVR exposure, PAR + UVA caused a consistent stimulation in the activity of their Arctic homologs. Natural PAR-driven stimulation of *Roseobacter* activity was commonly found in the Mediterranean, but seldom in the Arctic (Figure 4). *Bacteroidetes* showed a general lack of response upon exposure except in Antarctic samples, and *Gammaproteobacteria* presented more variable effects, showing a certain degree of PAR-stimulation coinciding with the highest abundances of the potentially photoheterotrophic NOR5 subgroup (Ruiz-González et al., 2012f).

**Figure 5:**
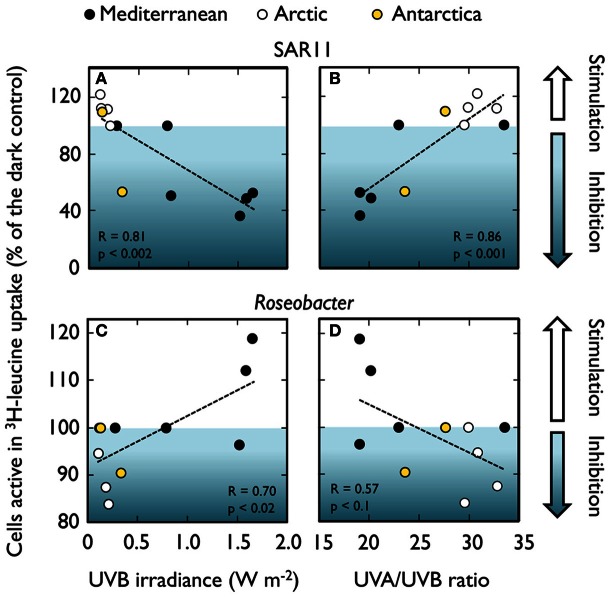
**Trends in responses to sunlight of bacterial groups from distinct habitats.** Relationships between sunlight-driven changes in the number of cells active in ^3^H-leucine uptake caused by full sunlight exposure (expressed as % of a dark control) and the UVB irradiances or the UVA to UVB ratio received by the samples in two subgroups of *Alphaproteobacteria*: the SAR11 clade **(A,B)** and *Roseobacter* **(C,D)**. Mediterranean data from Alonso-Sáez et al. (2006) and Ruiz-González et al. (2012f); Arctic and Antarctic values from Ruiz-González et al. (2012a).

The generalized lack of effects in winter and autumn in the Mediterranean (Figure 4) suggests that radiation levels during these seasons are too low to inflict damage that is detectable with this single-cell approach. In most of the cases, though, group-specific responses could not be directly related to the sunlight levels or any other measured environmental variable. Only when pooling SAR11 and *Roseobacter* data from both Mediterranean and polar waters (Figure 5) significant correlations arose between their responses and UVB irradiances (or the UVA to UVB ratio). As mentioned above, these two groups displayed opposite behaviors despite belonging to the same class. The scarcity of data available, though, precludes making generalizations about the responses of phylogenetically broad bacterial groups the different regions of the solar spectrum. The application of less used methods such as the PCR-DGGE combined with immunocapturing techniques, which allows the simultaneous identification of sequences of cells synthesizing DNA and the accumulation of thymine dimmers, could offer great potential for screening natural communities for UVR-resistant and sensitive bacterial phylotypes (Kataoka et al., 2009).

The observed light-driven increases in the activity of some bacterial phylotypes (see Figure 4) suggest that, besides sensitive or tolerant species, there may also be photoheterotrophic bacteria within a community. It has been suggested that photoheterotrophy is a rather common and widespread feature among aquatic bacteria (Karl, 2002), and three major types of photoheterotrophic prokaryotes have been described in aerobic aquatic environments: proteorhodopsin (PR)-containing bacteria, bacteriochlorophyll *a* (Bchl*a*)-containing bacteria (so-called aerobic anoxygenic phototrophs, AAPs), and picocyanobacteria capable of osmoheterotrophic uptake of DOM (see references Moran and Miller, 2007; Zubkov, 2009).

**Figure 6:**
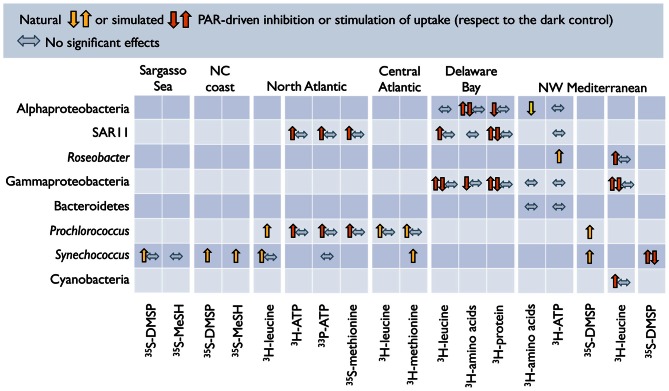
**Effects of PAR (natural or simulated) on the uptake of various radiolabeled organic substrates by different bacterioplankton groups as identified by MAR-CARD-FISH or flow cytometry cell sorting.** Arrows indicate whether PAR-stimulation, inhibition, or no effects were observed in various experiments done in the Sargasso Sea and the North Carolina (NC) coast (Malmstrom et al., 2005), the North Atlantic (Michelou et al., 2007; Gómez-Pereira et al., 2013), the Central Atlantic (Mary et al., 2008a), the Delaware Bay (Straza and Kirchman, 2011), and the NW Mediterranean (Alonso-Sáez et al., 2006; Vila-Costa et al., 2006; Ruiz-González et al., 2012b,e). The PAR-driven effects observed by Alonso-Sáez et al. (2006) and Ruiz-González et al. (2012b) on substrates other than leucine (see Figure 4) are included even though they also tested UVR radiation effects in their experiments.

Observed enhancements of activity are generally greatest under PAR exposure, typically decreasing or disappearing when UVR is included (Alonso-Sáez et al., 2006; Ruiz-González et al., 2012f; Figure 4). Studies focusing exclusively on the effects of visible light on the uptake of organic substrates are more abundant throughout the recent literature (Figure 6). For example, the use of flow cytometry cell sorting has shown that PAR may enhance the incorporation of various organic substrates by *Synechococcus* and *Prochlorococcus* (Malmstrom et al., 2005; Vila-Costa et al., 2006; Michelou et al., 2007; Mary et al., 2008a; Gómez-Pereira et al., 2013) but also by heterotrophic bacteria, presumably including SAR11 (Michelou et al., 2007; Mary et al., 2008a; Gómez-Pereira et al., 2013). Similarly, MAR-CARD-FISH revealed that exposure to PAR can increase the uptake of diverse labile substrates by *Synechococcus*, SAR11, *Roseobacter*, and *Gammaproteobacteria*, amongst others (Malmstrom et al., 2005; Straza and Kirchman, 2011; Ruiz-González et al., 2012e). However, such stimulation responses are not always consistent within a group and appear to vary seasonally (Straza and Kirchman, 2011; Ruiz-González et al., 2012f) or among sampling sites (Malmstrom et al., 2005).

Even though many of these studies have attributed photostimulation to the presumed occurrence and activity of photoheterotrophic taxa, we cannot conclude whether it was due solely to photoheterotrophy. As stated above, many processes occur under enhanced sunlight and increases in bacterial production caused or contributed by indirect effects cannot be discarded. Indeed, the benefits of light harvesting for bacteria are not well-understood and thus far we lack estimates of how the presence of photoheterotrophs may influence bacterial production measurements under light conditions. Whereas an increased uptake of leucine under visible light has been shown with cultured cyanobacterial strains (Chen et al., 1991; Mary et al., 2008b), this has not yet been demonstrated for representatives of the other two photoheterotrophic groups.

Very few studies have reported evidence for physiological advantages of PRs to marine bacterial isolates, namely promotion of growth (Gómez-Consarnau et al., 2006) or survival (Gómez-Consarnau et al., 2010). In contrast, light did not stimulate growth neither in a flavobacterial isolate (Riedel et al., 2010) nor in a cultured representative of the ubiquitous SAR11-encoding PR (Giovannoni et al., 2005). Under carbon starvation, though, the latter strain substituted light-mediated ATP production for endogenous carbon respiration (Steindler et al., 2011).

As for AAP bacteria, studies with isolates have reported (1) higher glucose uptake rates in alternate light-dark regimes than in continuous light or continuous darkness (Cooney et al., 2006), (2) positive effects of PAR upon starvation conditions, varying among isolates with different Bchl*a* content (Biebl and Wagner-Döbler, 2006), and (3) a substitution of respiratory carbon with photosynthetically driven ATP production (Koblízek et al., 2003). More recently, the first analysis of the transcriptional response of a cultured AAP to changing light revealed important roles of light in gene regulation (Tomasch et al., 2011).

All the aforementioned experiments with photoheterotrophic bacteria were conducted using only PAR, and none considered the effects of UVR. Whether photoheterotrophs are more resistant to full sunlight than strict heterotrophs remains unknown, and little is known about how they all behave and compete in natural sunlit environments. Evidences of increased PR expression upon light exposure in natural samples suggest an active role of these bacteria in aquatic ecosystems (e.g., Lami et al., 2009; Poretsky et al., 2009). However, observations such as the lack of competitive advantage of photoheterotrophs in summer compared to winter Arctic waters (Cottrell and Kirchman, 2009), or their increasing abundances toward lower transparency in estuarine waters (Waidner and Kirchman, 2007), suggest we are far from fully understanding the role that sunlight plays in the ecology of these photoheterotrophic strategists.

In summary, the potential effects of sunlight on bacterioplankton taxa are so diverse that predicting community behavior from compositional and taxon-specific physiological data is a formidable challenge. In some instances, though, taxonomy-resolved observations indicate that bulk bacterioplankton responses to light are largely driven by group-specific behaviors. For example, the PAR-driven stimulation of bulk ^3^H-leucine incorporation rates in Pacific and Atlantic waters was mostly attributed to *Prochlorococcus* (Church et al., 2004, 2006; Michelou et al., 2007; Mary et al., 2008a) or to these cyanobacteria plus certain groups of heterotrophic bacteria (Mary et al., 2008a; Gómez-Pereira et al., 2013). Low abundance *Gammaproteobacteria* were responsible for the photostimulation of bulk bacterial activity in Mediterranean waters exposed to simulated high PAR (Ruiz-González et al., 2012e). Similarly, the consistent inhibition of bulk ^3^H-leucine uptake observed in Mediterranean waters under full sunlight conditions (Alonso-Sáez et al., 2006; Ruiz-González et al., 2012f) was attributed to the dominance of UVR-sensitive SAR11. These studies point to a promising venue toward a better understanding of the roles of microbes in carbon cycling from their taxon-specific responses to environmental forcing.

Finally, the exploration of metagenomic data is unveiling a widespread distribution of photosensory proteins amongst aquatic bacteria (Singh et al., 2009). Various cellular functions such as pigment formation, DNA repair, stress responses, and the formation of biofilms or fruiting bodies are known to be mediated by light through different types of photoreceptors (Elías-Arnanz et al., 2011; van der Horst et al., 2007). This suggests that light may be influencing bacterial physiology in many other unexpected and generalized ways which deserve further investigation.

## Role of light exposure history

Besides explaining to some extent the community responses to current irradiance conditions, the composition of bacterial assemblages itself may also reflect the previous *in situ* light regime depending of the ability of communities for photoadaptation. Some recent studies have provided evidence that the *in situ* sunlight exposure history of the sampled bacterial assemblage may strongly determine the outcome of short-term experiments where the exposure is manipulated. When exposing samples to natural sunlight, most experimentalists take into consideration the radiation levels characteristic of a certain region or time of the year. Because of obvious experimental limitations, though, they generally use static incubations that neglect the critical role of water vertical mixing. Since the penetration of sunlight into the water column is wavelength dependent, mixing modulates the intensity, and spectral quality of the radiation to which the organisms are exposed at a time, and makes it change dynamically over time. As a consequence, deep mixing allows for recovery and photorepair at high UVA:UVB ratios after damage suffered at the higher UVB:PAR ratio of the surface. Hence, mixing depth and velocity, along with the optical properties of the system into consideration (Helbling et al., 2012), determine the dynamics of damage and repair, the acclimation of bacteria to their light regime, and their eventual responses to sunlight manipulation experiments.

Very few studies have attempted to examine the effects of mixing on the responses of bacteria to sunlight. Jeffrey et al. (1996a) reported dramatic differences in the vertical distribution of bacterial DNA damage depending on whether calm or strongly mixed conditions prevailed. Similarly, surface-incubated samples showed much more acute damage than samples collected after natural *in situ* mixing (Boelen et al., 2001). Huot et al. (2000) developed a model of UV-induced DNA damage in marine bacterioplankton, and observed that ozone thickness and the mixed layer depth are the most important factors driving the net DNA damage within the mixed layer. In accordance with these findings, the only report so far directly assessing the effect of mixing on bacterial heterotrophic activity shows that simulated mixing conditions greatly reduced inhibition of ^3^H-leucine incorporation rates in comparison to the average of samples exposed at various fixed depths, particularly in comparison to the inhibition of samples incubated at the surface, i.e., overexposed to UVB (Bertoni et al., 2011). Recent results of Galí et al. (accepted) also show that bacterial production values in bottles being vertically moved throughout the surface mixed layer resemble those statically incubated at the bottom of the mixed layer, stressing the role of vertical mixing in disrupting near-surface inhibition.

Overcoming the need for experimentally mimicking vertical mixing, other studies have provided indirect evidence for the importance of dynamic acclimation by comparing experimental conditions with the previous *in situ* light levels. For example, the magnitude of the PAR-driven increase in bacterial activity was found to be lower in assemblages sampled from highly irradiated surface waters, yet the reasons behind this observation were not explored (Straza and Kirchman, 2011). Similarly, PAR-driven inhibition of ^3^H-leucine and ^35^S-DMSP uptake was found to augment toward increasing overexposure of samples relative to their natural PAR conditions (del Valle et al., 2012). However, accurate calculations of the in situ light regimes must consider both meteorological and water mixing data. With this approach, bacterial production measured in sunlit incubations was found to be positively related to the previous UVR exposure, which was attributed to indirect effects on DOM (Xenopoulos and Schindler, 2003). A strong sunlight inhibition of bacterial activity in winter samples from a well-mixed water column, apparently caused by the experimental overexposure of these samples to UVB, was observed by Ruiz-González et al. (2012f). Likewise, the relative contribution of UVA and UVB to the inhibition of bulk ^3^H-leucine incorporation rates correlated to the previous *in situ* UVB:UVA ratio, suggesting that bacteria naturally acclimated to low UVB radiation levels were more sensitive to these short wavelengths in static incubations (Ruiz-González et al., 2012e). The same study reported that, over the year, the stimulation of bacterial activity by a constant level of artificial PAR increased toward greater differences between experimental light and the mean PAR within the mixed layer around noon. All this suggests that the observed overall responses of bacteria largely depend on how different the incubation conditions are from the natural light conditions the microorganisms are acclimated to; therefore, keeping the experimental exposure the most similar to *in situ* conditions is deemed necessary for an accurate interpretation of the observed responses. More research in this direction is needed to assess how the chosen exposure conditions may have affected activity measurement estimations in the studies conducted hitherto.

These light history-dependent responses would suggest that heterotrophic bacteria acclimate relatively fast (less than a day) to changing light conditions, yet conflicting results in the literature obscure this assumption. Although evidence of photoadaptation to UVB and UVA in cultured bacteria has been observed on some occasions (Joux et al., 1999; Berney et al., 2007), many studies with natural samples revealed no differences between the sensitivity of bacteria from high- and low-light environments (Bailey et al., 1983; Herndl et al., 1993; Xenopoulos and Schindler, 2003; Agogué et al., 2005; Hernández et al., 2007). Other studies, instead, have suggested that harsher exposure may result in acclimation or selection for more resistant assemblages (Thomson et al., 1980; Fernández-Zenoff et al., 2006; Joux et al., 2009; Bullock and Jeffrey, 2010; Santos et al., 2011b). Indeed, some recent experiments with bacterial isolates have shown that carotenoid-containing strains are more resistant to UV irradiation than non-pigmented cells (Dieser et al., 2010), yet shading due to high concentration of pigment in the suspensions of bacteria used might have overestimated the protective role of carotenoids.

It is possible that all these discrepancies are explained by the composition of the bacterial assemblages and the differential acclimation or resistance capabilities of taxa within them. In turn, the adaptation potential and rate of communities have been suggested to depend on the range of temporal and spatial variation in the environmental conditions to which they are naturally exposed (Wallenstein and Hall, 2012). Therefore, an accurate understanding of the dynamics in the bacterial responses to sunlight within and among communities through space and time will not be achievable without considering the scales of sunlight variability in aquatic ecosystems.

## Scales of variability in the exposure of aquatic microbes to sunlight

The quality and intensity of solar radiation received and perceived by a single planktonic cell fluctuates following changes in the solar zenith angle, but also in the depth range and intensity of the mixing processes, the attenuation in the water column, the cloud cover, and the presence of ice and snow at the water surface. All this translates into fluctuations on both temporal and spatial scales across which bacterial responses to sunlight are also expected to vary. However, few studies have taken into account these spatial and temporal scales of variability, so caution should be exerted when deriving conclusions or extrapolating from single experiments. In this section, we explore how the responses to sunlight of heterotrophic bacteria may change across these different scales.

### Spatial variability: changes through latitude, distance from shore, and depth

As a direct consequence of solar elevation, sunlight levels markedly decrease as one moves from the tropics toward the poles, so that the organisms inhabiting different latitudes are subjected to very different light regimes. The few large-scale studies available illustrate that there is latitudinal variation in the responses of bacteria to sunlight. Beyond the irradiance gradient, this variability has been attributed to the presence of different bacterial communities at different latitudes (Pakulski et al., 2007), or to the abundances and activity of particular groups of photoheterotrophs (Michelou et al., 2007; Mary et al., 2008a; Gómez-Pereira et al., 2013). Even though making a direct comparison of studies conducted at different latitudes is problematic due to methodological differences, the few data available show similar degrees of inhibition of ^3^H-leucine and ^3^H-thymidine incorporation by full sunlight in surface waters from a subtropical coral reef (Pakulski et al., 1998), the E Pacific (Pakulski et al., 2007), and Antarctica (Pakulski et al., 2008). More methodologically comparable experiments revealed similar inhibition percentages of bacterial production by full sunlight between a high mountain lake and the N Adriatic Sea (Sommaruga et al., 1997), and among Arctic, Antarctic, and NW Mediterranean waters (Ruiz-González et al., 2012a,f). At the single-cell level (Figure 4), it is remarkable that while almost no Mediterranean bacterial group showed responses to sunlight in winter, polar bacteria exposed to equally low UVB irradiances (see Figures 5A,C) did react to exposure, maybe indicating a greater susceptibility of polar organisms to sunlight.

At a smaller spatial scale, variable responses to sunlight have been found in shorter transects crossing different water mass characteristics. For instance, bacterial production in oligotrophic marine waters was inhibited by sunlight to a greater extent than in coastal waters influenced by the discharge of less transparent nutrient-rich freshwater (Joux et al., 2009). By contrast, no clear patterns were observed in bacterial responses to UV exposure despite the differences in optical and chemical properties along a transect from estuarine to offshore waters (Yuan et al., 2011).

Bacterial responses are also expected to vary through the water column due to the vertical gradient in radiation intensity and spectrum. By incubating samples at fixed depths, several authors observed that photoinhibition of bacterial activity decreased with depth along with radiation intensity (Aas et al., 1996; Lindell and Edling, 1996; Sommaruga et al., 1997; Pakulski et al., 1998; Morán et al., 2001; Visser et al., 2002), and that the spectral dependence of inhibition also varied with depth (Conan et al., 2008; Joux et al., 2009). In contrast, Church et al. (2006) found a vertical pattern of PAR-driven stimulation of bacterial activity that did not parallel that of PAR penetration. They attributed this discrepancy to the different heterotrophic capabilities of high-light and low-light adapted *Prochlorococcus*, which inhabited different depths of the water column. However, none of these studies considered vertical mixing, which, as already stated, has the effect of modulating the light field to which the organisms are actually exposed.

### Temporal variability: through days and seasons

Large variability in the light field also occurs over time. Throughout the day, aquatic microorganisms are exposed to changing conditions ranging from strong irradiances to darkness, with periodicities that evolve as we move to higher latitudes where extremes in day and night length occur.

The observations of significant diel variations in the bacterial incorporation of ^3^H-leucine and ^3^H-thymidine have been attributed to coupling with primary production (Fuhrman et al., 1985; Gasol et al., 1998; Shiah, 1999), release of DOM by grazers (Ruiz-González et al., 2012c), competition with phytoplankton for nutrients (Kuipers et al., 2000), viral infection (Winter et al., 2004), or direct damage by UVR and the associated repair processes (Jeffrey et al., 1996b; Pakulski et al., 1998, 2008; Walczak, 2008), among other mechanisms. It is thus likely that the responses of exposed communities vary depending on the time of sampling, just as many of the ecosystem process do. Since these short-term changes have sometimes been shown to overwhelm the annual variability (e.g., Ruiz-González et al., 2012c), caution should be exerted when deriving conclusions from punctual observations.

In addition, the few studies that have examined the photobiological role of different regions of the solar spectrum throughout the day indicate that the contribution of the different wavebands to bacterial inhibition also changes with time (Visser et al., 1999, 2002). On the other hand, how the activity of different heterotrophic bacterial groups, clades, or species varies at the diel scale remains largely unexplored. The only two reports to date show contrasting results: whereas no clear diel cycles in growth of three bacterial taxa were observed in North Sea waters (Pernthaler and Pernthaler, 2005), major bacterial groups from the NW Mediterranean were found to behave synchronously, showing higher activity at night (Ruiz-González et al., 2012c). In accordance with the latter observation, a comparative day/night metatranscriptomic analysis of North Pacific microbial communities revealed diel patterns of differential gene expression, including a greater nighttime abundance of heterotrophic bacterial transcripts related with amino acid acquisition and conversion (Poretsky et al., 2009).

Direct and indirect effects of solar radiation may also influence bacterial activity over large seasonal gradients. Irradiance levels increase from winter to summer and this, together with the shallower stratification of warmer waters, leads to an increased sunlight exposure of the organisms confined in the thinner surface layer. However, there is still a remarkable dearth of underwater light attenuation measurements through seasons and, again, most studies derive conclusions from occasional experiments conducted mostly in spring or summer. Bailey et al. (1983) were the first to report seasonal trends in the effects of UVR on natural bacterioplankton assemblages. Maximum inhibition of amino acid uptake in estuarine bacteria was observed in summer, whereas no significant effects were detected in winter. Moreover, they found that the spectral dependence of inhibition varied over time. At a shorter scale (<1 month) and with higher sampling resolution, bacterial production in a coastal lagoon was found to be either enhanced or inhibited by simulated PAR (Renaud et al., 2005), these responses being associated to variations in the phytoplankton assemblages and, probably, to the lability of the DOC they released. When the responses of Mediterranean bacteria to different wavebands were followed at a monthly frequency during two and a half years, the inhibition of bulk bacterial activity, mainly mediated by UVA, was highest during spring and summer and lowest in winter (Ruiz-González et al., 2012e). In the same study, removal of the natural variability of sunlight by incubating all samples under the same PAR intensity yielded seasonality in the responses, which was attributed to the presence of changing bacterial assemblages with different sensitivities. This hypothesis was built upon the observations of Alonso-Sáez et al. (2006), who incubated samples from the NW Mediterranean in spring and summer under different light treatments, and suggested that the observed stronger UVR inhibition of *Gammaproteobacteria* in spring might point to selection for photoresistant taxa in summer. However, the expected higher sensitivity of winter and fall bacterial assemblages could not be corroborated when these experiments were repeated throughout seasons (Ruiz-González et al., 2012f).

## Implications for carbon flux studies

Given that sunlight modulates the quantity and direction of carbon fluxes throughout the microbial food webs in so many ways, it stands as a key environmental factor to take into account when we are to make accurate estimations of these fluxes. It is true that sunlight effects are constrained to the illuminated layer of aquatic ecosystems (the photic zone), but it is also true that most autotrophic carbon production and more than half of the total prokaryotic heterotrophic production occur in this zone (Longhurst et al., 1995; Arístegui et al., 2009). In addition, surface waters mediate the exchanges with the atmosphere and most of the exchanges with neighbor land, thereby playing a key regulatory role in the rapid mobilization of carbon and elements among the different environmental compartments. Therefore, the magnitude of sunlight effects on aquatic heterotrophs is of relevance for both ecosystem and global carbon fluxes.

**Figure 7:**
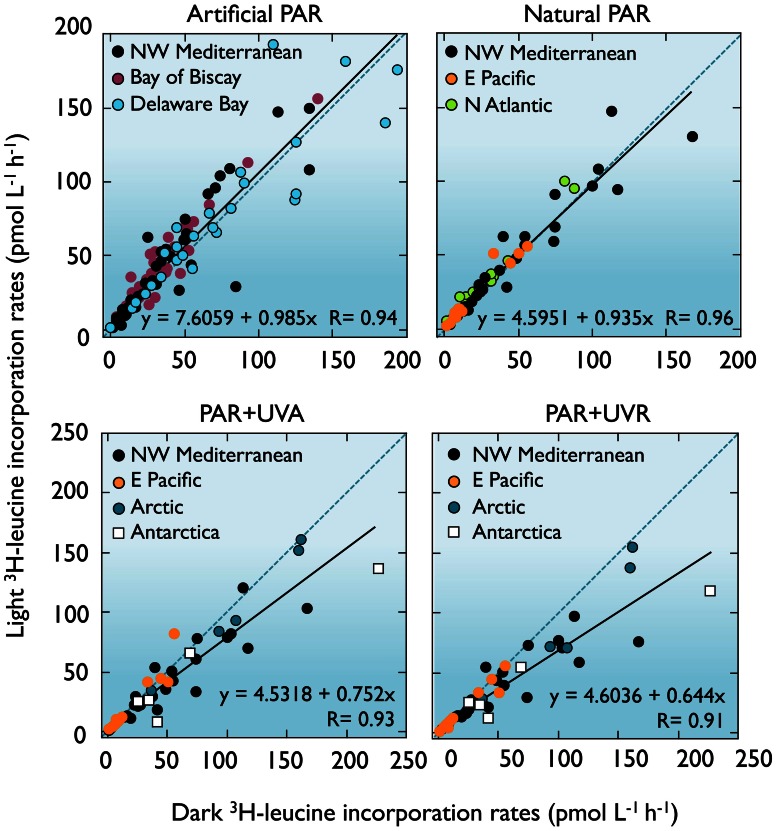
**Dark vs. light bacterial activity measurements.** Comparison between bulk ^3^H-leucine incorporation rates in different light conditions and dark incubations conducted with surface seawater samples (<5 m) from different systems and with the radiotracer added before exposure. Exposure to simulated PAR caused an average 23% stimulation while PAR+UVR led to an average 20% inhibition in comparison to the dark controls (see text). Mediterranean data from Ruiz-González et al. (2012e); Bay of Biscay (Calvo-Díaz, 2008); Delaware Bay (Straza and Kirchman, 2011); N Atlantic (Michelou et al., 2007); E Pacific (Pakulski et al., 2007); Arctic and Antarctica (Ruiz-González et al., 2012a and unpublished).

Much of our current understanding of bacterial heterotrophic activity and biomass production in aquatic systems is derived from ^3^H-leucine and ^3^H-thymidine incorporation measurements done in the dark. This approach, in view of the arguments developed above, may significantly over- or underestimate *in situ* bacterioplankton heterotrophic activity depending on the entangled processes simultaneously influenced by light. Upon compilation of comparable bacterial activity measurements from various aquatic systems done under different light conditions (Figure 7), a trend emerges: exposure to PAR + UVA and PAR + UVR leads to significant decreases in the slopes of the regression lines compared to the 1:1 line (ANCOVA, *F* = 32.6 and *F* = 70.2, respectively, *p* < 0.001), the strongest inhibition being caused by full sunlight (average 80 ± 22% of the dark control). Instead, even though no significant change in slope was apparent under natural or artificial PAR exposure, the higher Y-intercept of the simulated PAR fit line (*F* = 15.21, *p* < 0.001) indicates that this treatment significantly increases bacterial activity measurements (123 ± 38% of the dark control). Between the two extremes, an average 40% difference illustrates the relevance of the experimental light conditions in influencing the observed bacterial production estimates.

Likewise, most studies of primary production in aquatic systems are based on measurements conducted in the absence of UVR or under artificial light. To explore how carbon flows from phytoplankton to heterotrophic bacteria, both primary and bacterial heterotrophic production measurements should be made under comparable conditions. There is a large body of literature on the effects of UVR on primary production, in which a wide variability, similar to that encountered for bacteria, is reported: the effects range from strong to null inhibition, or even some stimulation, depending not only on the spectral quality and dose, but also on the species composition and light history of the algal assemblage, as well as on environmental factors such as temperature or nutrient limitation (see Villafañe et al., 2003 and references therein). Although bacteria are believed to be more sensitive to UVR than phytoplankton due to their small size and lack of efficient photoprotection (García-Pichel, 1994; Jeffrey et al., 1996a), the few studies that have evaluated simultaneously the effects of sunlight on primary and bacterial production show inconclusive (for contrasting) results: while some found that bacterioplankton production was more inhibited by UVR than primary production (Bertoni and Callieri, 1999; Sommaruga et al., 1999; Plante and Arts, 2000; Yuan et al., 2011), the opposite was also observed (Wickham and Carstens, 1998; Ferreyra et al., 2006; Ogbebo and Ochs, 2008). Overall, it seems that, even though the short-term effects of UVR on bacterial production may be stronger than on primary production, when integrated over time and space the activity of bacteria is less inhibited by UVR because it is sustained also during dark periods (Moran and Zepp, 2000). However, the amount of research to date is too limited to make reliable projections of autotrophy:heterotrophy shifts with changing regimes of sunlight intensity and spectrum (but see Godoy et al., 2012).

Sunlight has the potential to modulate the efficiency by which DOM is incorporated into biomass instead of being respired, the term named bacterial growth efficiency. Similarly to the case of bacterial production, most determinations of bacterial respiration are conducted in the dark. Results regarding the effects of sunlight on bacterial respiration are scarce and uncertain: while some authors found bacterial respiration rates (or numbers of actively respiring bacteria) to be inhibited by sunlight (Pakulski et al., 1998; Alonso-Sáez et al., 2006; Ruiz-González et al., 2012f), others found PAR or UVR-driven stimulation (Pakulski et al., 1998; Cottrell et al., 2008; Pringault et al., 2009; Hörtnagl et al., 2011), with contradictory consequences for the growth efficiency. Hence, although it is difficult to reach a consensus as to the actual effects of sunlight on bacterial respiration, the assumption that light and dark respiration rates are equivalent (and so are the growth efficiencies) should be rejected. To further complicate the picture, the effects of UVR on the respiration of several freshwater bacterial isolates also vary among strains, resulting in either increased or decreased bacterial growth efficiencies (Hörtnagl et al., 2011).

In view of the above, prediction of the ecosystem response to changes in the light regime is far from straightforward. Besides the stratospheric ozone reduction, global change associated shifts in the amount and optical properties of aerosols and clouds, air pollution, sea ice cover, surface reflection, upper stratification, and underwater light attenuation (e.g., by changes in DOC concentration), will all affect the doses of solar radiation in surface water bodies (Kerr et al., 2003; McKenzie et al., 2007). Some experiments in lakes have shown that the drought created by warmer air temperature led to increased acidification and reductions in water supply and DOC load, which resulted in increased water transparency to UVR (Schindler et al., 1996; Yan et al., 1996). Other studies in subarctic lakes, conversely, suggest that UVR transparency might decrease due to warming-derived increases in the density of forest cover and the associated terrestrial CDOM inputs (Pienitz and Vincent, 2000). There is also some evidence that the tendency for warmer sea surface temperatures may influence the timing and strength of stratification (Young and Holt, 2007). Should this lead to prolonged shallower stratification, marine organisms entrapped in the ocean surface would be exposed to increased radiation levels. However, mesocosm experiments simulating the expected increase in temperature and UVB radiation in the Mediterranean concluded that the largest effects on plankton communities were caused by warming and not by UVB enhancement nor by the combination of both stressors (Vidussi et al., 2011; Fouilland et al., 2013).

Overall, the available studies illustrate a large diversity in the mechanisms driving the ultimate response of bacterioplankton to sunlight, and it is the relative contribution of each of these mechanisms under different environmental scenarios what is likely to influence the paths of carbon flowing through aquatic food webs. These difficulties set a limit to our predictive capabilities upon changes in light regimes. Models of carbon flow within microbial food webs are getting more and more complex (see references in Gasol et al., 2008), yet they still do not include UVR, and hardly consider PAR among the environmental factors that control the dynamics of the heterotrophic components of aquatic ecosystems. Future research in this direction should comprise better characterizations of underwater PAR and UVR attenuation profiles, a deeper understanding of how taxonomically different bacterial communities perform under irradiation, and experiments addressing the synergies between UVR and other stressors simultaneously on various trophic levels. Of particular importance is the achievement of realistic experimental exposure conditions. If mimicking vertical mixing is not feasible due to logistic complexity, it is strongly encouraged that at least comparisons between the experimental and the previous *in situ* light regime are done to minimize misinterpretation of the obtained results. Finally, the development of experimental approaches encompassing the different temporal and spatial scales of light variability seems unavoidable if we are to scale up from short-term simple experiments to complex natural systems.

### Conflict of interest statement

The authors declare that the research was conducted in the absence of any commercial or financial relationships that could be construed as a potential conflict of interest.

## Appendix

**Table A1:** **Compilation of the existing literature on the effects of environmental levels of natural or simulated sunlight on bacterioplankton heterotrophic activity measured as of ^3^H-leucine or ^3^H-thymidine incorporation rates in natural samples**.

**References**	**Location**	**Light source**	**Light treatments**	**Time of exposure**	**Main effects (% of dark treatment)**	
					**Leucine Minimum**	**Thymidine Maximum**
**MARINE SYSTEMS**						
Carlucci et al., 1985	Baja California	Sunlight	PAR	1 h	100	
			PAR + UVR		100	
Herndl et al., 1993	N Adriatic Sea	Lamp	UVB	0.5 h	35–**129**	37–**144**
		Sunlight	PAR + UVA	4 h	59	
			PAR + UVR	4 h	9–15	
Müller-Niklas et al., 1995	N Adriatic Sea	Sunlight	PAR + UVR	4 h		15
Aas et al., 1996	Gulf of Mexico	Sunlight	PAR	1–11 h	61–**125**	60–**128**
			PAR + UVA		50–**120**	54–**123**
			PAR + UVR		34–**117**	25–**111**
Kaiser and Herndl, 1997	N Adriatic Sea	Lamp	UVB	2–4h	65–79	65–79
		Sunlight	PAR + UVR	3 h	61	52
Sommaruga et al., 1997	N Adriatic Sea	Sunlight	PAR	4 h	69	61
			PAR + UVA		31	46
			PAR + UVR		38	45
Pakulski et al., 1998	Pickles Reef (Florida)	Sunlight	PAR + UVR	48 h (12 h)	6–100	7–**164**
Chróst and Faust, 1999	Atlantic Barrier Coral Reef					
	Coastal lagoon	Sunlight	PAR + UVR	6 h	53–71	77–79
	Mangrove zone	Sunlight	PAR + UVR	6 h	46–55	51–57
Shiah, 1999	Kuroshio (Taiwan)	Sunlight	PAR	24 h (3 h)		100–**470**^*^
		Lamp	PAR	4–6 h		65–100
Visser et al., 1999	Caribbean Sea	Sunlight	PAR	3 h	81	
			PAR + UVA		47	
			PAR + UVR		47	
			PAR	8 h (2–3 h)	66–**130**	60–70
			PAR + UVA		47–100	50–70
			PAR + UVR		32–62	35–60
Gustavson et al., 2000	Gullmar Fjord (Sweden)	Sunlight	Respect to PAR + UVA	6, 11 days (2–4 days)		(% of PAR + UVA)
			PAR + UVR			100–**128**
		Sunlight + UVB lamp	PAR + UVR + UVB +			100–**169**
Morán et al., 2001	NW Mediterranean	Sunlight	PAR	2 h	23–100	
		Lamp	PAR		22–**528**	
	N Atlantic	Sunlight	PAR	3–6 h	33–**132**	
Visser et al., 2002	Caribbean Sea	Sunlight	PAR + UVR	8.5 h (3 h)	13–**134**	21–**128**
Church et al., 2004	North Pacific	Lamp	PAR	1–2 h	100–**392**	
		Sunlight	PAR	12 h	100–**336**	
Renaud et al., 2005	New Caledonia SW Lagoon	Lamp	PAR	1 h		72–**113**
Alonso-Sáez et al., 2006	NW Mediterranean	Sunlight	PAR	4 h	17–100	
			PAR + UVA		40–100	
			PAR + UVR		21–100	
Church et al., 2006	North Pacific	Sunlight	PAR	11–13 h	100–**192**	35–100
Hernández et al., 2006	Coliumo Bay (Chile)	Sunlight	PAR	11 h (3–4 h)	32–**112**	45–**123**
			PAR + UVA		19–75	24–74
			PAR + UVR		4–83	29–49
Michelou et al., 2007	N Atlantic	Sunlight	PAR	6 h	100–**340**	
Pakulski et al., 2007	E Pacific	Sunlight	PAR	4 h	100–**154**	30–**298**
			PAR + UVA		82–**146**	30–**295**
			PAR + UVA(>370 nm)		100–**164**	12–**273**
			PAR + UVR		56–100	9–**147**
Calvo-Díaz, 2008	Bay of Biscay (N Spain)	Lamp	PAR	1.5–2 h	49–**341**	
Conan et al., 2008	New Caledonia SW Lagoon	Sunlight	PAR	6 h		71–100
			PAR + UVA			53–100
			PAR + UVR			25–100
Pakulski et al., 2008	Palmer Station (Antarctica)	Sunlight	PAR + UVA	12 h	51–100	32–100
			PAR + UVR		30–86	2–85
			PAR + UVR	24 h (4–6 h)	10–100	0–75
Rochelle-Newall et al., 2008	New Caledonia SW Lagoon	Sunlight	PAR	4 h		60–100
Joux et al., 2009	NW Mediterranean (low salinity waters)	Sunlight	PAR	9–10 h	100	100
			PAR + UVR		48–100	48–100
	NW Mediterranean (marine waters)		PAR		100	81–100
			PAR + UVR		37–100	12–71
Bullock and Jeffrey, 2010	Gulf of Mexico	Sunlight	PAR + UVR	4 h	72	
Bertoni et al., 2011	NW Mediterranean	Sunlight	PAR	6.5–7 h	78–100	
			PAR + UVA		55–70	
			PAR + UVR		32–50	
		Sunlight (Fixed depths)	PAR + UVR		32–100	
		Sunlight (Mixing)	PAR + UVR		74–100	
Yuan et al., 2011	N South China Sea	Sunlight	*Respect to PAR*	5 h	*(% of PAR)*	
			PAR + UVR		46–**118**	
del Valle et al., 2012	North Pacific	Sunlight	PAR	4 h	64–**240**	
Ruiz-González et al., 2012c	NW Mediterranean	Lamp	PAR	2 h	**150**–**235**	100–**386**
Ruiz-González et al., 2012f	NW Mediterranean	Sunlight	PAR	4 h	79–100	
			PAR + UVA		70–100	
			PAR + UVR		30–100	
Ruiz-González et al., 2012a	Arctic Ocean	Sunlight	PAR + UVA	9.5–12 h	100	
			PAR + UVR		66–100	
	Antarctic Ocean	Sunlight	PAR + UVA	7.6–8 h	60–100	
			PAR + UVR		52–68	
Ruiz-González et al., 2012e	NW Mediterranean	Sunlight	PAR	2–3 h	68–**160**	
			PAR + UVA		42–**136**	
			PAR + UVR		40–**138**	
		Lamp	PAR	2 h	35–**250**	
Fouilland et al., 2013	NW Mediterranean	Sunlight + UVB lamp	*Respect to PAR + UVR*			
			PAR + UVR + UVB +	10 days (1 days)		67–100
**ESTUARIES**						
Aas et al., 1996	St. Rosa Sound (FL, USA)	Sunlight	PAR	2–10 h	87–**128**	72–**106**
			PAR + UVA		85–100	71–100
			PAR + UVR		57–99	47–88
Ziegler and Benner, 2000	Laguna Madre (TX, USA)	Sunlight	PAR	1 h	100–**152**	
			PAR + UVR		100–**163**	
Chatila et al., 2001	St. Lawrence estuary (Québec)	Sunlight	*Respect to PAR + UVA*	7 days (4 h)		*(% of PAR + UVA)*
			PAR + UVR			100–**162**
		Sunlight + UVB lamp	PAR + UVR + UVB +			100–**158**
		Sunlight + UVB lamp	PAR + UVR + UVB++			100–**173**
Ferreyra et al., 2006	St. Lawrence estuary (Québec)	Sunlight + UVB lamp	*Respect to PAR + UVR*			
			PAR + UVR + UVB++	9 days (1 days)		49–100
Bullock and Jeffrey, 2010	Pensacola Bay (FL, USA)	Sunlight	PAR + UVR	4 h	79	
Straza and Kirchman, 2011	Delaware Bay (NJ, USA)	Lamp	PAR	6 h	67–**174**	
Santos et al., 2011a	Ria de Aveiro (Portugal)	Lamp	UVB	9 h (3 h)	0–6	
	Ria de Aveiro (Portugal)	Lamp	UVB	4 h	7–58	21–69
**FRESHWATER SYSTEMS**						
Amon and Benner, 1996	Amazon River	Sunlight	PAR + UVR	3 h	100	100
			PAR + UVR	3–10 h	19–**218**	
Lindell and Edling, 1996	Lake Kariba (Zimbabwe)	Sunlight	PAR + UVR	4 h	51–100	
Sommaruga et al., 1997	Gosenköllesee lake	Sunlight	PAR	3–4 h	70–100	68–100
			PAR + UVA		25–**150**	21–100
			PAR + UVR		22–**150**	18–100
Bertoni and Callieri, 1999	Maggiore Lake (N Italy)		*Respect to PAR + UVA*	4 h	*(% of PAR + UVA)*	
		Sunlight	PAR + UVR		100	
		Sunlight	PAR + UVR		37–100	
		Sunlight + UVB lamp	PAR + UVR + UVB+		27–100	
		Sunlight + UVB lamp	PAR + UVR + UVB++		2–13	
Sommaruga et al., 1999	Gosenköllesee lake	Sunlight	*Respect to PAR + UVA*	16 days (4–5 days)		*(% of PAR + UVA)*
			PAR + UVR			34–100
Carrillo et al., 2002	La Caldera lake (Spain)	Sunlight	PAR	1 h		**282–426**
			PAR + UVA			**368–379**
			PAR + UVR			63–**228**
Medina-Sánchez et al., 2002	La Caldera lake (Spain)	Sunlight	PAR	1 h		**220–560**
			PAR + UVA			**250–1515**
			PAR + UVR			62–**500**
Xenopoulos and Schindler, 2003	2 lakes (NW Ontario)	Sunlight	*Respect to PAR*		*(% of PAR)*	
			PAR + UVA	4 h	100	
			PAR + UVR		36 −100	
			PAR + UVA	48 h	100–**182**	
			PAR + UVR		100–**282**	
Medina-Sánchez et al., 2006	La Caldera lake (Spain)	Sunlight	PAR	1 h		**284–446**
			PAR + UVA			**382–396**
			PAR + UVR			63–**239**
Ogbebo and Ochs, 2008	Sardis reservoir (MS, USA)	Sunlight	*Respect to PAR*	7 days (2–5 days)	*(% of PAR)*	
			PAR + UVR		100	
Bullock and Jeffrey, 2010	Blackwater river (FL, USA)	Sunlight	PAR + UVR	4 h	57	
Santos et al., 2011a	Lake Vela (Portugal)	Lamp	UVB	9 h (3 h)	0–8	

The limits of the range of effects (minimum value–**maximum value**) are given as % of the dark control (or of any other light treatment when indicated). The 100% indicates no significant light-driven effect. Whether samples were exposed to natural sunlight or to artificial light sources is also indicated. Total exposure time is given, and numbers in brackets indicate the intervals, if any, at which samples were taken. Experiments where something else than light was manipulated (i.e. nutrient additions, temperature manipulations, pre-exposure of filtered water) were not included.

* The stimulation found by Shiah (1999) took place at night in transparent carboys which had been exposed to sunlight during the day, not exactly after exposure.
